# Editorial

**Published:** 1873-10

**Authors:** 


### Editorial.

### THE DENTAL LAW.

There seems to be some misunderstanding in regard to the effect of the amendment made to the dental law of Ohio by the last legislature. The whole matter will be apparent by a careful reading of the law as amended, which we herewith append. The law regards those who have been in practice five years and over, as having complied with its provisions, or at least are free from its requirements. But those who seek to enter the profession with less than five years practice, must comply with the requirements of the law, viz : have a diploma from some reputable dental college, or have passed a satisfactory examination by a board of examiners. It was feared by many that the amendment passed by the last legislature, would greatly lessen, if not destroy, the efficiency of the law for good. Though in a small degree in one direction, the law may fail to accomplish all that the most sanguine would desire, yet the great object to be accomplished by the law is not abated one iota. And the profession should now more than ever see to it, that its provisions are complied with by those who enter its ranks, or seek to do so.

The profession has it in their power to make the line of distinction between the thoroughly competent practitioner, and the ignorant, incompetent, so unmistakeably defined and apparent, that scarcely any one need be deceived. Those who enter the profession should have such attainments and abilities as to command attention and respect. The colleges and boards of examiners have great influence over these matters. The foundation of all these things is with the profession, and a prompt and decisive demand from this direction would not be disregarded.

The law as amended is as follows :

### A LAW TO REGULATE THE PRACTICE OF DENTISTRY IN THE STATE OF OHIO.

Amendment to section one of an act entitled : An act to regulate the practice of Dentistry, in the state of Ohio," passed May 8th, 1868.

SECTION 1. Be it enacted by the General Assembly of the State of Ohio. That section one (1) of the above named act be so amended as to read as follows : That it shall be unlawful for any person to practice Dentistry in the State of Ohio for compensation, unless such person has received a Diploma from the faculty of a Dental College duly incorporated under the laws of this or any other State of the United States, or foreign country, or a certificate of qualification issued by the State Dental Society, or by any local Society auxiliary thereto, provided that in all cases, where any person has been continuously engaged in the practice of dentistry for a period of five years or more, such person shall be considered to have complied with the provisions of this act, and the act to which this is amendatory.

SEC. 2. That said original section one (1) be and the same is hereby repealed.

SEC. 3. This act shall take effect and be in force from and after its passage.

Passed March 10th, 1873.

SECTION 2, 3 AND 4 OF ORIGINAL ACT.

SEC. 2. Any person who shall practice Dentistry without having complied with the regulations of this act, shall be deemed guilty of a misdemeanor, and upon conviction thereof, shall be fined not less than fifty dollars nor more than two hundred dollars ; provided, that nothing in this act shall be construed to prevent physicians and surgeons from extracting teeth.

SEC. 3. All prosecutions under this act shall be by indictment before the Court of Common Pleas in the County where the offense was committed, and all fines imposed and collected under the provisions of this act, shall be paid into the Treasury of the County where such conviction shall take place, for the use of the Common Schools within such County.

SEC. 4. This act shall take effect and be in force from and after its passage.

### DENTAL EDUCATION.

The subject of dental education is one that ought to elicit the highest interest of every dentist. Every one should feel the importance of the subject enough to examine the character and standing of our educational institutions, and seek, where there are defects, to have them corrected, and sustain those that are most efficient in their work.

Unfortunately there is a great want of interest in this matter, and in many cases not only a lack of interest, but an active opposition, more or less pronounced, either in word or act. Many dentists will discourage students, and often prevent them from entering a dental college; affirming that they will have better opportunities for obtaining the desired knowledge in an office. Others manifest an entire indifference as to whether their students take a college course or not, giving them little or no preparation for it, and manifesting little or no interest what institution, if any, the student enters.

For the erroneous and foolish views, so often entertained by students, in reference to the proper curriculum of study and work preparatory to practice, the practitioners who assume to be preceptors are responsible. We always dislike fault-finding, but when there is so much that could be easily done, that is not done, we sometimes become a little impatient. There are however, quite a variety of phases that attach to this matter. And as an illustration of the influences exerted to induce teachers and faculties to let down their just rules and requirements, which they in their best judgment have seen fit to establish, with a view to the best good of all concerned, we here give the main points of two letters just now come to hand.

"Sept. 13th, 1873.

Dear Doctor :—It will be impossible to be there more than half the time, and I want to know definitely, if possible, if more than half fees would be exacted. Unless there be some inducement, it seems hardly worth the while to go there, when I can be accomodated nearer home, and save traveling expenses. I feel the need of help, and would gladly stay through, but, situated as I am, cannot possibly more than half. Please write soon that I may know what to expect. *"

Another year and a half student.

"Can I graduate from your college, if I take one course of lectures If I can't, then I know I can elsewhere. *"

We have had many inquiries and propositions of a similar character, and have as often referred such applicants to the regulations of the college, with the statement "that the faculty declines to make any special arrangements, or make any special bidding or bargaining, as inducements for students." Any such special arrangements are always demoralizing to all concerned, and will invariably work injury.

There are other influences constantly operating to prejudice the best interests of professional education, such as an over-anxiety to secure large classes, and to graduate the largest number, irrespective of the character of the material composing the classes.

An emulation as to who shall accomplish the greatest good is always laudable ; a purpose is always best accomplished by doing thoroughly that which is attempted.

### ELEVENTH ANNUAL MEETING OF THE CONNECTICUT VALLEY DENTAL ASSOCIATION

Will be held at Haynes' Hotel, Springfield, Mass., Tuesday and Wednesday, Oct. 21–22. The Executive Committee invite attention to the following

ORDER OF EXERCISES :

Reading of Records.
Admission of New Members.
Reports of Treasurer, and Publishing Committee.
Election of Officers.
Retiring President's Address.
Induction of Officers.

SUBJECTS FOR DISCUSSION :

1st.—Essay, by George L. Parmele, M. D. Subject not announced.

2nd.—Sensitive Dentine, its connection with Neuralgia, or a Neuralgic Diathesis.

3d.—A case ofFractured Maxilla, (treated by Dr. Gunning, of New York.) Essay, by C. A. Brackett, D. M. D.

4th.—Failure of Teeth Filling.

5th.—Building up of Teeth worn away by Mastication.

6th.—Has experience proved the expediency of Deep Chambers, and Artificial Ridges?

7th.—Miscellaneous Subjects.

Holyoke, Mass., Sept. 23, 1873. L. C. TAYLOR, *Sec'y.*

### A NEW FILE CARRIER.

We have used for a few days a file carrier invented and constructed by Dr. Bowman, of Columbus, O., that is superior in some respects to any other carrier we have seen. It is very simple in construction, is not easily broken or disarranged, it holds any thin file very firmly, and a thick file is easily adapted to it; there is no tremor or springing of the file while being used. The file runs parallel with the handle. This is a valuable instrument, and should be in the hands of every operator. They will soon be in the market.

### DENTAL ASSOCIATION.

At the annual meeting of the San Francisco Dental Association, held Monday, Sept. 8th, 1873, the following officers were elected for the ensuing year: *President*, Dr. S. W. Dennis *Vice President*, Dr. H. Austin; *Corresponding Secretary*, Dr. H. E. Knox; *Recording Secretary*, Dr. S. E. Knowles; *Treasurer*, Dr. J. J. Birge. The President, on taking the chair, refreshed the memories of the members in a few happy remarks on the work that had been accomplished by associated efforts, and called their attention to the vast amount of work that could, and would be done in the future, by a unison of action between officers and members. The best of feeling prevailed.

